# Cytoplasmic incompatibility management to support Incompatible Insect Technique against *Aedes albopictus*

**DOI:** 10.1186/s13071-018-3208-7

**Published:** 2018-12-24

**Authors:** Riccardo Moretti, Giuseppe Augusto Marzo, Elena Lampazzi, Maurizio Calvitti

**Affiliations:** 1 Biotechnology and Agroindustry Division, ENEA (Italian National Agency for New Technologies, Energy and Sustainable Economic Development), Casaccia Research Center, Rome, Italy; 2 Technologies and Facilities for Nuclear Fission and Nuclear Material Management, ENEA (Italian National Agency for New Technologies, Energy and Sustainable Economic Development), Casaccia Research Center, Rome, Italy

**Keywords:** *Wolbachia*, AR*w*P, bidirectional incompatibility, risk assessment, SIT, population suppression, population replacement

## Abstract

**Background:**

The transinfection of the endosymbiotic bacterium *Wolbachia* provides a method to produce functionally sterile males to be used to suppress mosquito vectors. AR*w*P is a *w*Pip *Wolbachia* infected *Aedes albopictus* which exhibits a bidirectional incompatibility pattern with wild-types. We coupled a modelistic approach with laboratory experiments to test AR*w*P as a control tool and evaluate the possible occurrence of population replacement following the release of AR*w*P females in a wild-type (S_ANG_) population. Repeated male-only releases were simulated and tested in the laboratory in comparison with releases contaminated with 1% AR*w*P females. Model simulations also investigated how migration affects the outcome of contaminated releases. Finally, the mean level of egg fertility and the long-term evolution of populations constituted by two *Wolbachia* infection types were studied by testing S_ANG_ and AR*w*P *Ae. albopictus* and performing more general model simulations.

**Results:**

The model was parametrized with laboratory data and simulations were compared with results of biological trials. Small populations of AR*w*P males and females were theoretically and experimentally demonstrated to rapidly become extinct when released in larger S_ANG_ populations. Male-only releases at a 5:1 ratio with respect to the wild-type males led to a complete suppression of the S_ANG_ population in a few generations. Contaminated releases were efficient as well but led to population replacement in the long term, when the wild-type population approached eradication. Migration significantly contrasted this trend as a 5% population turnover was sufficient to avoid any risk of population replacement. At equal frequencies between AR*w*P and S_ANG_ individuals, the mean egg fertility of the overall population was more than halved and suppression was self-sustaining until one of the two infection types extinguished.

**Conclusions:**

In the case of bidirectional incompatibility patterns, the repeated release of incompatible males with small percentages of contaminant females could lead to population replacement in confined environments while it could be managed to target high efficiency and sustainability in wild-type suppression when systems are open to migration. This possibility is discussed based on various contexts and taking into consideration the possibility of integration with other control methods such as SIT and the use of larvicides.

**Electronic supplementary material:**

The online version of this article (10.1186/s13071-018-3208-7) contains supplementary material, which is available to authorized users.

## Background

*Aedes albopictus* (Skuse) (Diptera: Culicidae), the Asian tiger mosquito, is responsible for an increasing public concern due to its rapid worldwide spread and to its potential role as vector of severe diseases [1, 2]. In various cases, this species showed a major role in supporting epidemics in the tropics, mainly due to its relative abundance [3, 4]. In addition, the remarkable capability to produce overwintering eggs proved to increase the risks of epidemics related to this vector in temperate climate areas [5], as demonstrated by the dengue [6, 7] and chikungunya [8, 9] virus outbreaks that occurred in Europe in recent years.

Decades of extensive use of insecticides led to the development of mosquito populations resistant to several active ingredients [10, 11] compromising effectiveness and sustainability of this traditional control method in the long-term [12]. Consequently, various alternative control strategies are under experimentation to reduce the epidemiological role of mosquitoes [13]. Among the most innovative strategies, the genetic control approaches aim to suppress or eliminate vector populations or replace them with ones showing reduced fitness or decreased vector competence [14, 15]. Specifically, these methods are mainly based on the release of sterile males obtained by irradiation [16], transgenesis [17] or by the transinfection of the endosymbiotic bacterium *Wolbachia* [18].

*Wolbachia* is a widespread endosymbiotic bacterium of arthropods and nematodes which induces significant modifications in the reproductive biology of its hosts thus promoting the spread of the infection in conspecific uninfected populations [19].

The horizontal transfer of *Wolbachia* from donor insects to both uninfected and already infected species has been widely reported as reviewed previously [20]. Besides the objective of biological studies, the purpose of these attempts was the exploitation of certain host biological traits associated with *Wolbachia* infection to set up control strategies against insect pests and vectors [18]. In particular, a mechanism of post-mating sterility, named cytoplasmic incompatibility (CI), occurs when a spermatozoon from an individual infected by a specific *Wolbachia* strain fertilizes an uninfected egg or an egg infected by a further non compatible *Wolbachia* strain [19, 21, 22]. The CI pattern is bidirectional when matings between males and females harboring different *Wolbachia* strains are reciprocally incompatible. Instead, CI is unidirectional when the females belonging to one of the two infection types are fertile with both types of males. This latter CI pattern usually characterizes crosses between *Wolbachia*-infected and uninfected populations.

*Wolbachia*-based strategies for vector control are considered environmentally benign and cost effective since they are self-sustaining thanks to the maternal inheritance of the infection and the CI mechanism [23, 24]. The feasibility of transferring horizontally this bacterium between species is making the exploitation of *Wolbachia* a promising tool to produce functionally sterile males. In addition, an alternative control strategy can be supported by the property showed by certain *Wolbachia* strains to reduce the vector competence of newly infected mosquito species, as demonstrated with *Aedes aegypti* (Diptera: Culicidae) [25, 26], the main vector of dengue and Zika viruses (respectively, DENV and ZIKV). A small *Ae. aegypti* population harboring a double *Wolbachia* infection has been recently reported [27], however the species is generally not infected by *Wolbachia* in nature. The artificial introduction of a specific *Wolbachia* strain (*w*Mel from *Drosophila melanogaster*) led to the establishment of a laboratory line with reduced vector competence and capable of invading wild-type populations thanks to the unidirectional CI. This finding paved the way to innovative approaches to fight mosquito-borne viruses through the replacement of the vectors with conspecific populations incapable of supporting diseases [28].

As such, various models have been developed to ascertain efficacy and safety of *Wolbachia*-based approaches for vector control. These models were mainly addressed to study the effects of CI and simulate the conditions determining the spread of useful biological traits [29–35] or, more specifically, aimed to estimate how biological traits such as a *Wolbachia*-induced reduction in the vector competence may impact the transmission of severe diseases [36–39]. However, as noted by other authors [40], for theoretical predictions to have any practical validity, models have to be parametrized with field data and validated for the particular species of interest.

In 2008, a new *Aedes albopictus* line was established in ENEA Casaccia Research Center (Roma) through the replacement of the wild-type *Wolbachia* infection (*w*AlbA and *w*AlbB strains) with a *Wolbachia* strain caught from *Culex pipiens molestus* [41, 42]. The population, named AR*w*P, was characterized in the subsequent years and selected to reduce the fitness costs initially associated with the new infection [43]. Currently, AR*w*P shows favorable traits for field application as a suppression tool against *Ae. albopictus* [44]. In fact, AR*w*P males induce full CI throughout their entire life when mating with wild-type females and show at least equal mating competitiveness with wild-type males. Compared to *Ae. albopictus* irradiated at 30 Gy, AR*w*P males are capable of inducing a higher level of induced sterility both in small laboratory cages [45] and even more evidently under large enclosures in the field [46]. In this last context, they also competed significantly better than untreated *Ae. albopictus* individuals belonging to laboratory-reared and wild-caught populations. Furthermore, under the intense rearing protocols typical of the genetic control strategies [17] and compared to the wild-type *Ae. albopictus*, the time needed for AR*w*P emergence is significantly shorter and produced males per female are not significantly different [46].

Even if these biological traits seem to be favorable for field application, other considerations and studies are needed to ascertain the safety of the IIT approach against *Ae. albopictus* before moving to open field trials*.* In fact, based on the current unavailability of perfect methods for adult sexing, the risks associated to accidental, or practically unavoidable, releases of AR*w*P females should be investigated carefully [42, 47]. To do this, we made use of a model and carried out specific experiments aiming to: (i) ascertain the invasive potential of AR*w*P *Ae. albopictus* in a wild-type population and the risks of replacement; (ii) evaluate the efficacy of a suppression strategy based on the use of incompatible males against *Ae. albopictus* both when only-male or female-contaminated releases are performed; (iii) validate the model as a means to simulate the population dynamics of different coexisting *Wolbachia* infection types in *Ae. albopictus*; and (iv) develop the opportune strategy for *Ae. albopictus* suppression, concurrently aiming at best efficacy, lower risk, higher sustainability.

## Methods

### *Aedes albopictus* populations and rearing conditions

Two mosquito lines were used in the experiments. S_ANG_ originated from wild-type *Ae. albopictus* individuals from Anguillara Sabazia (Rome) collected in 2006 and since then reared under laboratory conditions at ENEA-Casaccia Research-Center (Rome). AR*w*P is a CI-inducing line, established at ENEA-Casaccia Research Centre in 2008 through the transinfection of *Wolbachia*-cured S_ANG_ individuals with *w*Pip *Wolbachia* from *Culex pipiens* [41] and reared for about 100 generations under rearing settings described below. Both the above described lines were periodically outcrossed with wild-type individuals from the same area to preserve the genetic variability [43].

Larvae were brought to adulthood inside 0.5 litre larval trays at the density of 1 larva/ml, augmented with a powder obtained by crushing dry cat food (Friskies® Adults) at a fixed dose of 4 mg/larva of which 10% was given on day 1, 45% on day 2 and 45% on day 5. Adult mosquitoes were kept inside 40 × 40 × 40 cm cages at T = 28 ± 1 C°, RH = 70 ± 10 %, L:D = 14:10 hours and were supplied with water and sucrose. Following previous authors [48], the blood meals were offered by a thermostated blood feeder filled with defibrinated fresh swine blood heated at 38-39 °C, for 1 hour.

### The model

The model that we used to study *Ae. albopictus* population dynamics in response to the presence of different *Wolbachia* infection types builds upon that presented by other authors [29]. The model was revised, and a number of additional parameters were taken into consideration allowing for a greater flexibility in reproducing the specific experimental system and in simulating the expected dynamics. Since our aim was to simulate the effect of an incompatible males release referring to a specific case (AR*w*P vs. wild-type *Ae. albopictus*), various parameters have been specifically adjusted based on laboratory data.

Based on the above model, if *N(t)* is the population of female mosquitoes at time *t*, the expected female progeny at time *t + 1*, referring to a single homogeneous mosquito population, results from equation 1. In this first equation, *m* stands for mean female fecundity (i.e. the mean number of produced L_1_ daughters) and *S*_*N*_ is the density dependent survivorship determining the proportion of first instar larvae developing to adult females capable of reproducing again (Eq. 2).

1 $$ {N}_{t+1}={N}_t{mS}_N $$

2 $$ {S}_N=\frac{S_0}{1+{\left(\alpha {N}_{t+0.5}\right)}^{\gamma }} $$

*S*_*N*_ results from the mean individual survival in the absence of intraspecific competition (*S*_0_), the type of intraspecific competition (*γ*) (generally supposed to occur only at the larval stage in mosquitoes, [49]), the number of first instar larvae (*N*_*t+0.5*_) and a constant (*α*) related to the carrying capacity of the population [50, 51]. The type of intraspecific competition can be portrayed as contest competition (*γ* = 1; when available resources are utilized only by one or a few individuals) or scramble competition (*γ >* 1; when resources are accessible to all of the individuals) [29]. The variables and their corresponding values related to the insect species are summarized in Table 1.

**Table 1:** Variables used to simulate the population dynamics which were common to the two *Ae. albopictus* populations as not related to the *Wolbachia* infection type. Data are specifically referred to S_ANG_ (= *Y* infection type) and AR*w*P (= *X* infection type) *Ae. albopictus*

Variables	Description	Calculation	Values		
			Based on literature	Measured in laboratory	Set in experiments and model simulations^c^
*m*	Female fecundity (Mean number of daughters produced by a female)	To be set based on the mean viable eggs produced per blood meal or long life		19.88 ± 1.12	20 ± 2
*S*_*0*_	Immature survival in absence of intraspecific competition (%)	To be set based on laboratory data	~̴20^a^	84.91 ± 1.12	depending on the experiments
*γ*	Competition type (=1 if contest, >1 if scramble)	Estimated based on literature	>1^b^		1.5
*α*	Constant relative to the carrying capacity of the population	Estimated based on the experimental system	Depending on the context	~̴̴ 0.0002	0.0002

^a^Results regarding *Ae. albopictus* in nature [58]

^b^Value obtained from literature [29]

^c^Specific values were attributed to the variables to run the model as corresponding to the mean values obtained from previous research or specific experiments involving S_ANG_ and AR*w*P *Ae. albopictus*

*Abbreviations*: *S*_*ANG*_ Wild-type *Ae. albopictus* with natural *Wolbachia* infection, *ARwP w*Pip *Wolbachia-*infected *Ae. albopictus*

*Wolbachia* is known to affect further parameters (Table 2) which can have significant effects on the reproductive biology of a population. When two infection types (*X* and *Y*) are concurrently present in a population, they may cause effects that directly influence the overall population dynamics together with the frequencies of the two infection types at the following generations. Based on literature related to *Aedes* sp., these effects are mainly mediated by the *Wolbachia* strain-specific influence on: female fecundity (*F* factor, with *F*_*X*_ = *F*_*Y*_ = 1 when the *Wolbachia* strain does not significantly affect fecundity, [52, 53]); larval survival (*L* factor, with *L*_*X*_ = *L*_*Y*_ = 1 when the *Wolbachia* strain does not significantly affect larval survival, [54]); egg mortality (*H* factor, representing the proportion of viable eggs produced by an incompatible cross; when the *Wolbachia*-mediated incompatibility is complete, *H* = 0) [42].

**Table 2:** Variables used to simulate the population dynamics which were related to the *Wolbachia* infection type of the two *Ae. albopictus* populations. Data are specifically related to S_ANG_ (= *Y* infection type) and AR*w*P (= *X* infection type) *Ae. albopictus*

Variables	Description	Calculation	Values		
			Based on literature	Measured in laboratory	Set in experiments and model simulations
*Hx*	Percent egg hatch in crosses between *X* females and *Y* males	Based on laboratory data and literature		2.56 ± 0.02^a^	3.00
*Hy*	Percent egg hatch in crosses between *X* males and *Y* females	Based on laboratory data and literature	0.00^b^	0.00	0.00
*Fx*	Fecundity costs determined by the *X* infection	Based on laboratory data		1	1
*Fy*	Fecundity costs determined by the *Y* infection	Based on laboratory data		1	1
*Lx*	Costs on immature survival by the *X* infection	Based on laboratory data and literature	1^c^	1	1
*Ly*	Costs on immature survival by the *Y* infection	Based on laboratory data and literature	1^c^	1	1
*q*_*X*_	Frequency of the aposymbiotic males produced by the *X* females	Based on laboratory data	0^c^	0	0
*q*_*Y*_	Frequency of the aposymbiotic males produced by the *Y* females	Based on laboratory data	0^d^	0	0
*C*_*Mx*_	*X* Male competitiveness in comparison with *Y* males	Based on laboratory data		1.24^e^	1.20
*C*_*My*_	*Y* Male competitiveness in comparison with *X* males	Based on laboratory data		0.76^e^	0.80

*S*_*ANG*_ Wild-type *Ae. albopictus* with natural *Wolbachia* infection, *ARwP w*Pip *Wolbachia* infected *Ae. albopictus*

^a^See Additional file 2: Table S2

^b^According to previous data [44]

^c^According to previous data [41]

^d^According to literature [80]

^e^See Additional file 3: Table S3

Thus, the amount of *X* and *Y* females at the *n+1* generation results, respectively, from Equations 3 and 4, with *a*, *b*, *i*, and *j* standing for the frequencies of *X* females, *Y* females, *X* males, and *Y* males respectively. *μ* stands for the fraction of uninfected eggs produced by the *X* and *Y* females and *q*_*t*_ represents the frequency of expected aposymbiotic males (based on *μ* value) which would be fertile when mating with both *X* and *Y* females. When *μ* = 0, obviously also *q*_*t*_ = 0.

3 $$ {N}_{X_{t+1}}={N}_{X_tm}\left({a}_t\left(1-{\mu}_X\right){F}_X\right)\left({i}_t+{j}_t{H}_X+{q}_{Xt}+{q}_{Yt}\right)\frac{S_0{L}_X}{1+{\left(\alpha {N}_{X_tm}\left({a}_t\left(1-{\mu}_X\right){F}_X\right)\left({i}_t+{j}_t{H}_X+{q}_{Xt}+{q}_{Yt}\right)\right)}^{\gamma }} $$

4 $$ {N}_{Y_{t+1}}={N}_{Y_tm}\left({b}_t\left(1-{\mu}_Y\right){F}_Y\right)\left({j}_t+{i}_t{H}_Y+{q}_{Xt}+{q}_{Yt}\right)\frac{S_0{L}_Y}{1+{\left(\alpha {N}_{Y_tm}\left({b}_t\left(1-{\mu}_Y\right){F}_Y\right)\left({j}_t+{i}_t{H}_Y+{q}_{Xt}+{q}_{Yt}\right)\right)}^{\gamma }} $$

#### Assumptions

A series of assumptions aimed at simplifying the study without significantly affecting the validity of the simulation but allowing to focus our attention on key parameters only associated with the *Wolbachia* infection type. These assumptions regard parameters which are not expected to differ in dependence of the *Wolbachia* strain based on the available literature. Specifically, we assumed that: (i) all individuals were virgin at start; (ii) both the *X* and *Y* populations were characterized by a 1:1 sex ratio (kept as a constant over the tested generations); (iii) distribution of the infection types and matings were random; (iv) generations were not supposed to overlap; (v) population growth was stage-structured; (vi) the experimental system was confined; and (vii) the vertical transmission of both the infection types was perfect, leading to the absence of aposymbiotic individuals [41]. All of these assumptions are controllable under laboratory settings while they have to be taken into consideration when discussing the possible outcome of similar experiments in open field conditions.

Because it is known that temperature, food availability, and larval density may affect development, adult dimensions, and fitness [55–57], these parameters have been kept constant throughout the bioassays.

#### Added variables

Further variables are additional with respect to previous models [29]. We introduced the possibility to choose whether to release a new stock of incompatible males (*X*) at each *n* generation or just once, at *n* = 0. The ratio (*R*) of *X* to *Y* males at releases can be defined based on the expected frequencies of *Y* males at each generation.

A female contamination factor (*U*) takes into account the ratio of *X* female contamination expected at each *X* male release. This factor allows to take into consideration the additional females which are not expected from the data regarding the infection type frequencies available at the previous generation. These females are not involved in competition at larval stage but can produce progeny and may affect the population dynamics at the following generations.

A male mating competitiveness factor (*C*_*M*_) accounts for the potential differences between infection types. When *C*_*M*_ = 1 in both infection types, male mating competitiveness does not depend on the infection type, while different values should be associated with not random matings.

A larval competitiveness factor (*C*_*L*_) can also be taken into consideration in the case that different infection types may cause a differential ability in exploiting available food causing long-term effects not directly correlated with survival. This parameter may take into account possible differences regarding time of immature development and sizes of adult females (directly influencing fecundity) and males (possibly correlated to the male mating competitiveness). If *C*_*LX*_*= C*_*LY*_*=* 1, larval competitiveness does not depend on the infection type. These features represent delayed density-related effects [49].

Following the addition of the above assumptions and added variables and referring, as an example, only to the *X* population, we have:

5 $$ {N}_{X_{t+1}}=\left({N}_{X_tm}\left({a}_t{F}_X\right)\left({i}_t{C}_{MX}+{j}_t{C}_{MX}{H}_X\right)\frac{S_0{L}_X{C}_{LX}}{1+{\left(\alpha \left(\left({N}_{X_tm}\left({a}_t{F}_X\right)\left({i}_t{C}_{MX}+{j}_t{C}_Y{H}_X\right)+\left({N}_{Y_tm}\left({b}_t{F}_Y\right)\left({j}_t{C}_{MY}+{i}_t{C}_{MX}{H}_Y\right)\right)\right)\right)\right)}^{\gamma }}\right)+{RUN}_{Y_{t+1}} $$

The frequencies of the *X* infected females (*a*) and males (*i*) in Equation 5 can be calculated also taking into account the *R* and *U* parameters which determine number and sex ratio of the *X* infected individuals to be added to the *X* population deriving from the previous generation.

This equation refers to a confined environment while an optional factor which takes into account migration will be added for a specific model simulation.

### Preliminary tests and set up of the parameters to run the model

Preliminary tests were carried out for determining specific factors capable to fit the model to the experimental system. In the case of availability, results obtained from previous experiments were also taken into consideration. First of all, we verified possible differences between S_ANG_ and AR*w*P *Ae. albopictus* with regards to: (i) mean fertile female eggs produced per female (*m*; estimated by assuming a 1:1 sex ratio); (ii) immature survival (S_0_). This investigation ascertained that these fitness parameters were not affected by the *Wolbachia* infection (Additional file 1: Table S1).

The fertility rate in CI crosses (*H*_*X*_ and *H*_*Y*_) and the factor accounting for the male mating competitiveness (*CM*_*X*_ and *CM*_*Y*_) were also compared and they are shown, respectively, in Additional file 2: Table S2 and Additional file 3: Table S3.

### Invasive potential of AR*w*P in wild-type populations

This experiment aimed at determining the capability of the AR*w*P infection type to increase its frequency in a S_ANG_ wild-type population.

S_ANG_ and AR*w*P 3-5 days old males were released in a large experimental cage (100 × 100 × 100 cm) at 5:1 ratio for a total of 60 males. A mixed population of 2-4 days old females (50 S_ANG_ and 10 AR*w*P) was then added. Mating was allowed for 24 hours and then a blood meal was made available. Produced eggs were collected on wet germination paper until 7th day after feeding and then hatched at 10th day. Regardless of the G_n_ and the number of individuals, larvae were reared based on the protocol reported above.

At each subsequent generation (G_n_) adults were allowed to emerge individually, and 60 females and 60 males were randomly selected to constitute a new experimental cage to be blood-fed for egg production. At each G_n_, 20 among the remaining individuals were analyzed by PCR to ascertain infection type frequencies. Five parallel repetitions were carried out.

Results were compared with those obtained by running the model on the basis of the parameters values defined in the first paragraph of the Results section.

### Population size under IIT application

A second experiment had the objective to analyze the population size dynamics of an *Ae. albopictus* population following a long-term IIT application, both in presence or absence of residual females among the males to be released.

At G_0_, the experimental cage (100 × 100 × 100 cm) was constituted by a mixed population of S_ANG_ and AR*w*P Ae. *albopictus* individuals based on the following ratios: 50:50:250 S_ANG_ males:S_ANG_ females:AR*w*P individuals. Males and females were aged as in the previous experiment. Two different treatments were tested. In IIT_i_ (ideal IIT), the AR*w*P population was constituted by male-only releases. In IIT_c_ (contaminated IIT), 1% of the AR*w*P individuals were females instead of males to take into account possible defects in the sexing procedure. In both cases and regardless of the infection type, females were added 1 hour after the males allowing them to mix. The same protocol reported in the previous experiment was followed with regard to blood meals and egg collection. At each G_n_, larvae were reared in a 200 ml rearing tray and food quantity per larva was kept constant, based on the proportions and protocol reported above. At each G_n_ the rearing tray was replaced.

Based on the fact that immature survival of AR*w*P and S_ANG_ *Ae. albopictus* were not found to significantly differ (see the results of the preliminary experiments) and also taking into consideration the expected immature survival of *Ae. albopictus* in nature [58], survival at each G_n_ was arbitrarily set at 20%. Consequently, each G_n_ experimental cage was constituted by a number of females “NF” = 0.1 × NL_1(Gn)_, randomly selected from all of the emerged, and kept virgin, females, with NL_1(Gn)_ standing for the total number of first instar larvae obtained from the eggs oviposited by the females of the G_n-1_. The above females were added to a mosquito population constituted by the same amount of males (NF) and by a number of newly introduced AR*w*P individuals in 5:1 proportion with respect to NF. In IIT_i_ test, all of the latter AR*w*P individuals were males, while, in IIT_c_ test, a 1% of contaminant females among males was again set. When needed, not integers were rounded up to the next integer to calculate the number of females to be added to replace AR*w*P males.

The end of the experiment was determined by the following events: the extinction of the caged population (possible in both IIT_i_ and IIT_c_ trials); the fixation of the released infection type (only possible in IIT_c_); or, otherwise, at G_7_. Both trials were replicated 5 times.

Results were compared with those obtained by running the model on the basis of the parameters values defined in the first paragraph of the Results section.

### The effect of migration on the possibility of population replacement under IIT_c_

We tested the effect of introducing in the model a factor accounting for the immigration and emigration of individuals when applying IIT_c_ to evaluate the conditions determining AR*w*P invasiveness in a non-confined environment. The population growth rate of the tested population (inner system) was supposed to be equal to that of the surrounding one (inhabiting the outer system). In the absence of control measures, the rates of immigration (IR) and emigration (ER) were assumed to be equal because inner and outer systems were considered in equilibrium. The numbers of immigrants (N_i_) and emigrants (N_e_) were supposed to vary proportionally to the population size based on the IR and ER values. Under these conditions, an IIT approach is expected to reduce the population growth rate and consequently the N_e_ value. Instead, N_i_ was assumed to be not affected by the control measures applied in the tested area as the outer system was considered unlimited. In particular, we simulated 5:1 IIT_c_ releases and tested the effect of different IR and ER values (0.00, 0.01, 0.02 and 0.05 proportions of the population size) on the possibility by AR*w*P to replace the S_ANG_ population in the tested area. Obviously, immigrants were expected to belong to the wild-type population. Instead, emigrants were supposed to be constituted by mixed infection types at proportions corresponding to their frequency in the inner system and assuming that the two infection types have the same ER. The population dynamics of the inner system were studied for 20 generations.

### Egg hatch and population dynamics when two incompatible infection types coexist

A further experiment aimed to measure the mean egg fertility in a mixed population of AR*w*P and S_ANG_ *Ae. albopictus* and also investigated the evolution of this experimental system in the short term of 5 generations.

At G_0_, AR*w*P:S_ANG_ ratio was set at 1:1 (25:25 virgin males added with 25:25 virgin females). The same protocol reported in the previous experiments was followed with regard to blood meals and egg collection. At egg hatch, the fertility level of the mixed G_0_ population was measured. Hatched larvae were reared to adulthood to constitute the following generation. At each G_n_, 50 females and 50 males were randomly selected among the adult individuals to constitute a new experimental cage and to be blood-fed for egg collection while 20 among the remaining individuals were analyzed by PCR to measure the variation of the AR*w*P frequency in the following generations. The experiment was repeated 5 times.

Two model simulations were also run to study the population dynamics of *Ae. albopictus* in the case of presence of two bidirectionally incompatible infection types. Both simulations started with an *Ae. albopictus* population constituted by 100 males and 100 females and the two infection types were supposed to not differentially affect host biology and behavior. In the first case, the proportions of the two infection types were set at 0.5 and the effect of varying the S_0_ value (0.1, 0.2, 0.3) on the population dynamics of the tested population was studied. The second simulation compared control cages where all of the individuals were infected by a single *Wolbachia* infection type with test cages where the frequencies of the two infection types were set at 45:55 to avoid the establishment of a stable equilibrium. Also in this case, the population dynamics were studied having in mind that control and test cages could have represented systems subjected, respectively, to IIT_i_ and IIT_c_ approaches at the moment of stopping releases.

### *Wolbachia* molecular diagnosis

PCR assays were performed to measure AR*w*P frequency in experimental trials using a specific set of primers: *w*PF (5'-CGA CGT TAG TGG TGC AAC ATT TA-3') and *w*PR (5'-AAT AAC GAG CAC CAG CAA AGA GT-3') capable of amplifying a *w*Pip *Wolbachia*-specific sequence of 281 bp [44]. DNA was extracted from individual mosquitoes by dissecting and homogenizing their abdomens in 100 μl STE with 0.4 mg/ml proteinase K. The PCR cycling procedure used was: 94°C for 5 min followed by 35 cycles of 94°C for 30 s, 54°C for 30 s, 72°C for 40 s and a single final step at 72°C for 10 min. Amplified fragments were electrophoresed on 1.5% agarose gels, stained with ethidium bromide (1 μg/ml) and visualized under ultraviolet light.

### Data analysis

The results from multiple experiments are reported as averages when appropriate. The adopted uncertainties are the standard deviations. The latter is a conservative choice with respect to the standard deviation of the mean which would potentially underestimate the uncertainties given the limited number of data averaged. Percent data related to immature survival were transformed to arcsin square root of proportions before analysis. Normality of the experimental data was determined by the Shapiro-Wilk test. One-way repeated-measures ANOVA was used to compare female fecundity and immature survival between the two *Wolbachia* infection types.

The above statistical analysis was performed using PASW statistics (PASW Statistics for Windows, Version 18.0. SPSS Inc., Chicago, USA).

As a measure of the goodness of the fit when the model results are compared to the experimental data, the reduced chi-square is adopted in the following analysis. The reduced chi-square of an ideal, perfect fit is 1. Large values are indicative of a poor fit, very small values suggest overestimation of the data uncertainties.

## Results

### Determination of the factors to be set in advance before the bioassays

A series of factors were determined based on previous research and/or literature to reduce the number of variables of the model and allowing for a more meaningful comparison with the biological trials reported in the following paragraphs. Apart from the first generations after the establishment of the line [41], AR*w*P did not show to significantly differ from S_ANG_ *Ae. albopictus* with regard to the female fecundity (*m* value) as confirmed in the present work (Additional file 1: Table S1). Using our experimental set up, *m* was calculated taking into consideration the overall studied female population (also including the females refusing the blood meal). As reported in Table 1, the obtained value exhibited an intrinsic range of variability which had to be taken into account when the model is compared to the experiments. The above results allowed to set both *F*_*X*_ and *F*_*Y*_ values at 1 (for simplicity, avoiding referring to the aposymbiotic line; Table 2).

We also previously demonstrated that immature survival did not significantly differ comparing AR*w*P and S_ANG_ [41] and this biological trait was stable in the following generations until present (Additional file 1: Table S1). Consequently, a single *S*_*0*_ value has been attributed to both populations and, accordingly to previous works showing a not significant impact by the *Wolbachia* infection type on immature performance [55], *L*_*x*_ and *L*_*y*_ were also set as both equal to 1, as reported in Tables 1 and 2. In Table 1, the *S*_*0*_ value obtained in laboratory is compared with a value verified to occur in nature [58], where the immature stages of *Ae. albopictus* also face predators, pathogens and often reduced resources. When running the model, we preferred to set a *S*_*0*_ value similar to that obtained in nature, in order to obtain a more realistic basic reproductive rate (the product of *m* and *S*_*0*_) [29] allowing for a better discussion of the possible results achievable in open field.

Estimating the *γ* value was not simple since the type of competition in mosquitoes is strictly dependent on density and, in addition, sex-specific [55]. We fixed it (Table 1) also based on the simulations of other authors [29] but being aware of the fact that defining a reliable *γ* value representing both mosquito males or females would have meant to only approximate reality. In fact, females require a higher amount of resources compared to males to reach pupal stage and males and precocious females may monopolize them in the case that they are a limiting factor. Furthermore, our studies mainly regarded larval populations at a density not expected to promote a strong competition for resources and, under these conditions, γ was expected to have a minor influence on the outcome of the model. In fact, it is worthwhile to note that, given its mathematical definition, the model is expected to be numerically insensitive to a large range of values for the *γ* (as far as *γ* > 1) and *α* parameters when the described populations are far from their carrying capacities. This is the case of our experimental system, therefore in the following experiments the other model parameters will not be influenced by approximate values for *γ* and *α* as also evidenced by Additional file 4: Figure S1. We also set an *α* value that we observed to fit with the carrying capacity of our experimental system (Table 1).

In Table 2, further values attributed to various parameters under *Wolbachia* influence are shown. The *H*_*X*_ values was measured by the authors and results from the product of the fertility rate obtained when AR*w*P females mate with SR males not harboring *w*AlbA infection [42] and the mean fraction of S_ANG_ males non harboring the *w*AlbA *Wolbachia* infection measured in our laboratories (Additional file 2: Table S2). Instead, *H*_*Y*_ is 0 [41].

Aposymbiotic individuals are not expected to occur in both AR*w*P [41] and S_ANG_ *Ae. albopictus*, thus *q*_*X*_ and *q*_*Y*_ values were 0.

Regarding male mating competitiveness (*C*_*M*_ factor), statistically significant differences were found between AR*w*P and S_ANG_ males as a Fried index of about 1.7 was measured for AR*w*P males in large enclosures (Additional file 3: Table S3). In this work, the *C*_*L*_ parameter, as defined in methods section, has been set to 1 since there are no data available from experiments carried out by us or from literature to define *Wolbachia*-related differences. Nevertheless, the possible effect of this parameter on the expected results will be analyzed in the Discussion section.

In Table 3, a series of variables is listed which were used for the set up of the experimental conditions and the model simulations described in the following paragraphs. The specific values attributed to each parameter were determined by the experimental conditions on the basis of equation 5.

**Table 3:** Variables which were defined depending on the experiment and used to simulate the population dynamics of *Ae. albopictus* when different incompatible *Wolbachia* infection types are present. Data are specifically related to S_ANG_ (= *Y* infection type) and AR*w*P (= *X* infection type) *Ae. albopictus*

Variable	Description	Calculation	Values		
			Based on literature	Measured in laboratory	Set in experiments and model simulations
*Nx*_*t*_	Number of *X* infected females at *t* = 0	Based on the ratio of female contamination at male releases			It depended on the experiment
*Qx*_*t*_	Number of *X* infected males at *t* = 0	Based on the ratio of incompatible males at releases			It depended on the experiment
*Ny*_*t*_	Number of *Y* infected females at *t* = 0	Set arbitrarily, based on the amount of the target population			It depended on the experiment
*Qy*_*t*_	Number of *Y* infected males at *t* = 0	Set arbitrarily based on the amount of the target population			It depended on the experiment
*a*_t_	Frequency of the released *X* females at *t* = 0	Measured based on the data above			It depended on the experiment
*i*_*t*_	Frequency of the released *X* males at *t* = 0	Measured based on the data above			It depended on the experiment
*b*_*t*_	Frequency of the released *Y* females at *t* = 0	Measured based on the data above			It depended on the experiment
*j*_*t*_	Frequency of the released *Y* males at *t* = 0	Measured based on the data above			It depended on the experiment
*U*	Proportion of contaminant *X* females at each X males release	To be set based on laboratory data	0.002^a^	0.002^a^	0.01
*R*	Ratio between released *X* and *Y* males at releases	To be set arbitrarily			It depended on the experiment

^a^Percentage of residual females when applying the Standard Operating Procedures of mechanical sexing on AR*w*P *Ae. albopictus* [46]

### Invasive potential of AR*w*P in wild-type populations

**Fig. 1:**
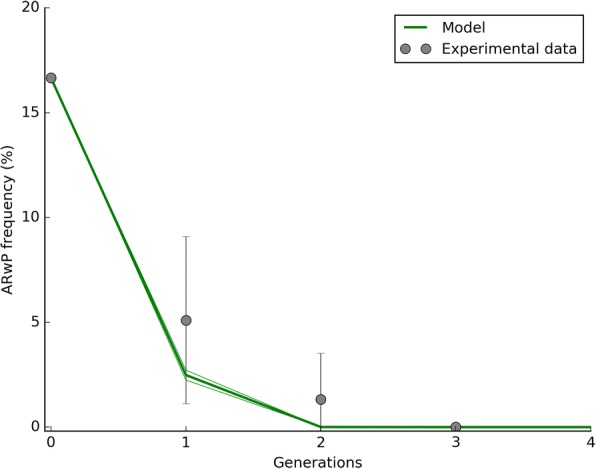
AR*w*P is unable to invade a wild-type *Ae. albopictus* population. The experiment started from from a ratio of 1:5 AR*w*P:S_ANG_ individuals and at 1:1 sex ratio. Due to the Bidirectional Incompatibility pattern, a fast decrease in the frequency of the AR*w*P infection type was observed in the following generations. Experimental data are compared with model predictions which also show a region of space illustrating the female fecundity (*m*) intrinsic variability. The reduced chi-square was 0.88

Data reporting on the capacity by AR*w*P *Ae. albopictus* to invade a wild-type population are represented by Fig. 1. Starting from 1:5 proportion between the two infection types, AR*w*P frequency dropped down to 0 in a few generations. As comparison, the model was run by using the parameters reported in Tables 1 and setting the parameters reported in Table 2 accordingly to the requirements of the experiment. The model outlined a similar decline of the AR*w*P infection type over a few generations (Fig. 1).

### Population size under IIT application

**Fig. 2:**
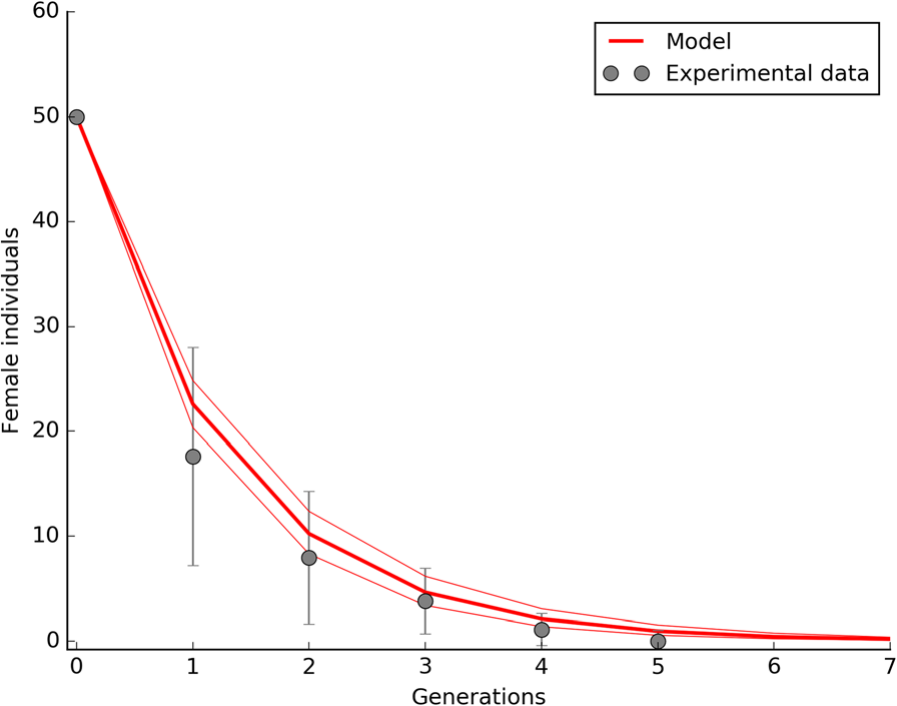
Suppression of a S_ANG_ *Ae. albopictus* wild-type population by the IIT_i_ approach. The IIT_i_ trials were characterized by the periodical release (a release for generation) of AR*w*P males at 5:1 ratio with S_ANG_ males. Under the tested experimental conditions the wild-type population was eradicated in a few generations. Experimental data are compared with model predictions which also show a region of space illustrating the female fecundity (*m*) intrinsic variability. The reduced chi-square for the IIT_i_ trials was 1.01

Under the experimental conditions of the IIT_i_ trial, a 5:1 ratio between released incompatible and wild-type males was sufficient to induce an efficient population suppression leading to the eradication of the *Ae. albopictus* population in five generations (Fig. 2). As shown in the same figure, the model predictions were observed to strictly confirm this trend.

**Fig. 3:**
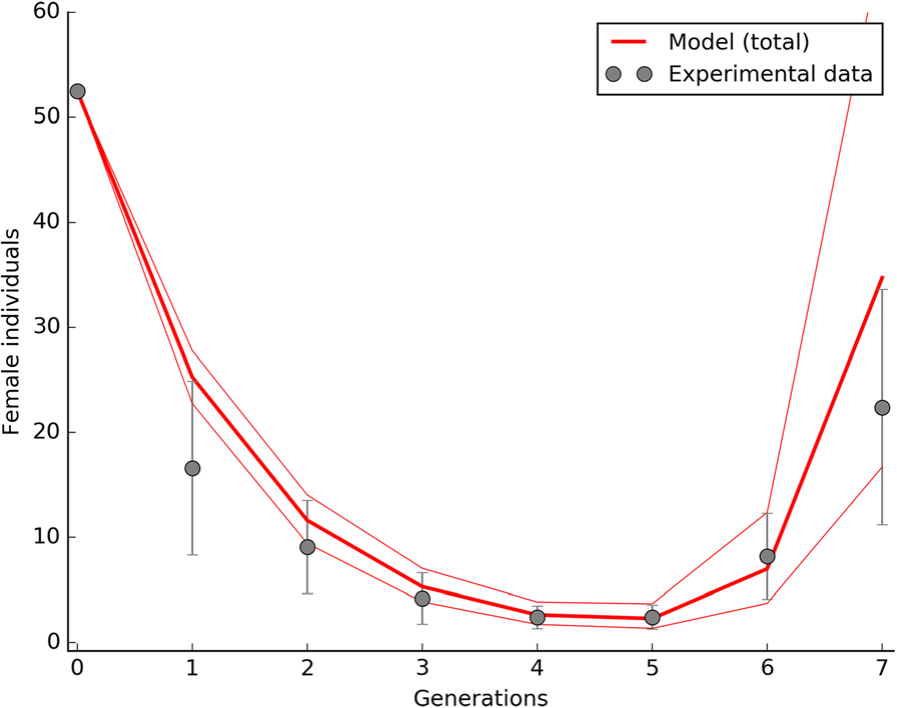
Suppression of a S_ANG_ *Ae. albopictus* wild-type population by the IIT_c_ approach. The IIT_c_ trials were characterized by the periodical release (a release for generation) of AR*w*P individuals at 5:1 ratio with S_ANG_ males. Male releases were 1% contaminated by AR*w*P females. Under the tested experimental conditions the wild-type population was rapidly suppressed however, in 1 case out of 5, the AR*w*P infection type replaced S_ANG_ and established, leading to the failure of the control strategy. Experimental data are compared with model predictions which also show a region of space illustrating the female fecundity (*m*) intrinsic variability. The reduced chi-square for the IIT_c_ trials was 1.04

With regard to the IIT_c_ approach, a substantial suppression of the wild-type population was obtained as well, however, in 1 out of 5 repetitions, the AR*w*P infection type invaded the wild-type population before its eradication leading to a population replacement and, consequently, to a failure of the control strategy. Mean experimental values are reported in Fig. 3 in comparison with the data obtained from the model which also predicts the risk of population replacement.

### The effect of migration on the possibility of population replacement under IIT_c_

**Fig. 4:**
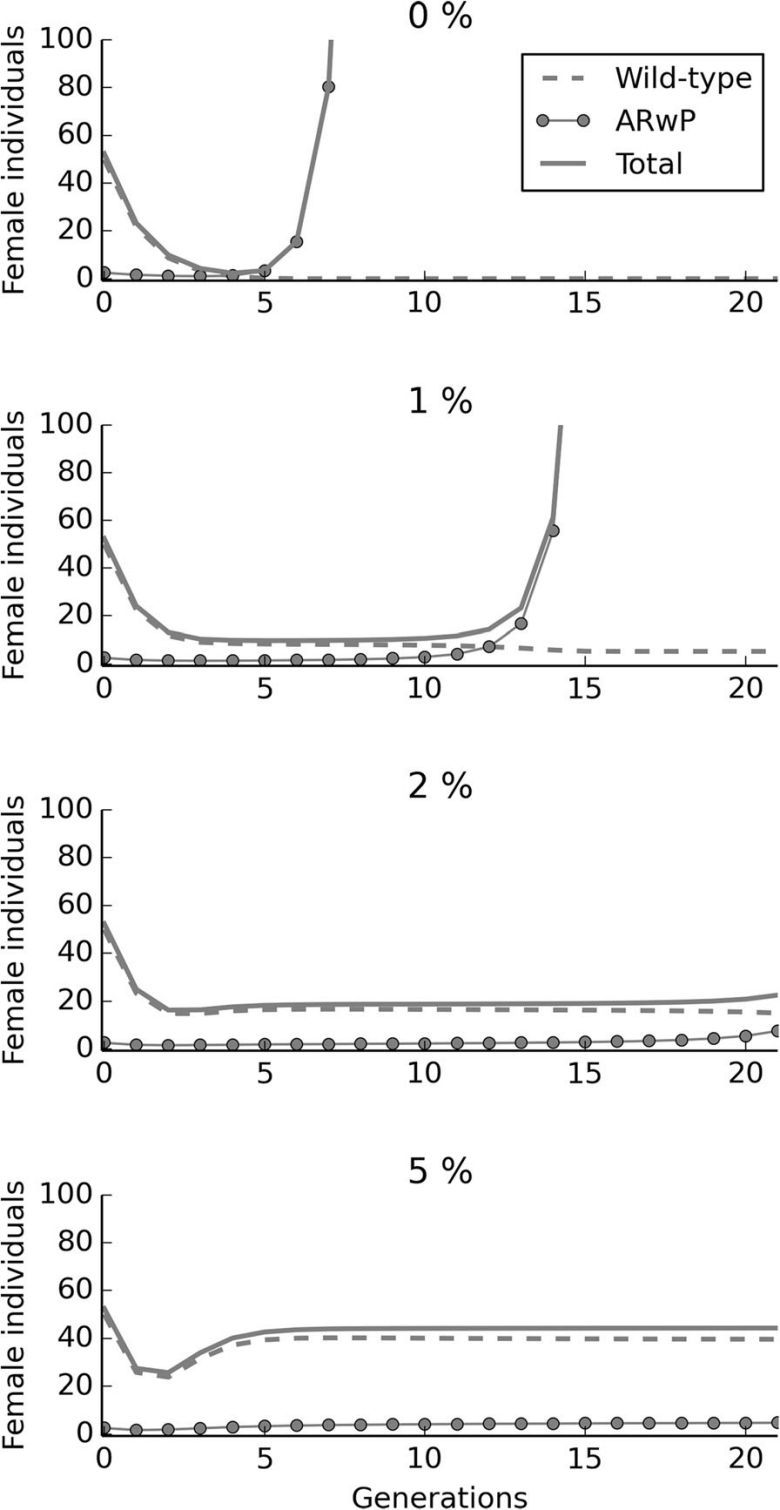
Simulation of the effects of migration on *Ae. albopictus* population dynamics and AR*w*P spreading when applying an IIT_c_ approach. The introduction of a factor accounting for the migration of individuals has a profound impact on the outcome of the model simulation of the IIT_c_. Increasing the percent population turnover (0, 1, 2, 5%) means gradually delaying the population replacement by AR*w*P of the wild-type *Ae. albopictus* population

Migration was found to significantly affect the outcome of IIT_c_. In fact, the model simulation demonstrated that a 1% turnover in the overall population subjected to repeated contaminated releases was sufficient to delay any risk of population replacement beyond ten generations since the start of the releases (Fig. 4). In the same figure it is also shown as a 5% turnover avoided replacement for more than 20 generations.

### Egg hatch and population dynamics when two incompatible infection types coexist

As expected, the mean egg fertility was significantly reduced by the coexistence of two incompatible *Wolbachia* infection types. Specifically, mean egg hatching was more than halved under the tested experimental conditions (respectively, 70.63 ± 9.84 and 24.50 ± 9.61 % in wild-type control and mixed populations), in accordance with results reported previously [46]. However, the system proved to be highly unstable as, on average, AR*w*P frequency gradually increased during the following generations. In fact, in 3 out of 5 cases, the AR*w*P infection type encountered fixation by G_4_, G_5_ was needed for the eradication of S_ANG_ *Ae. albopictus* in a 4th repetition and, in 1 case out of 5, AR*w*P extinguished and S_ANG_ was instead fixed.

**Fig. 5:**
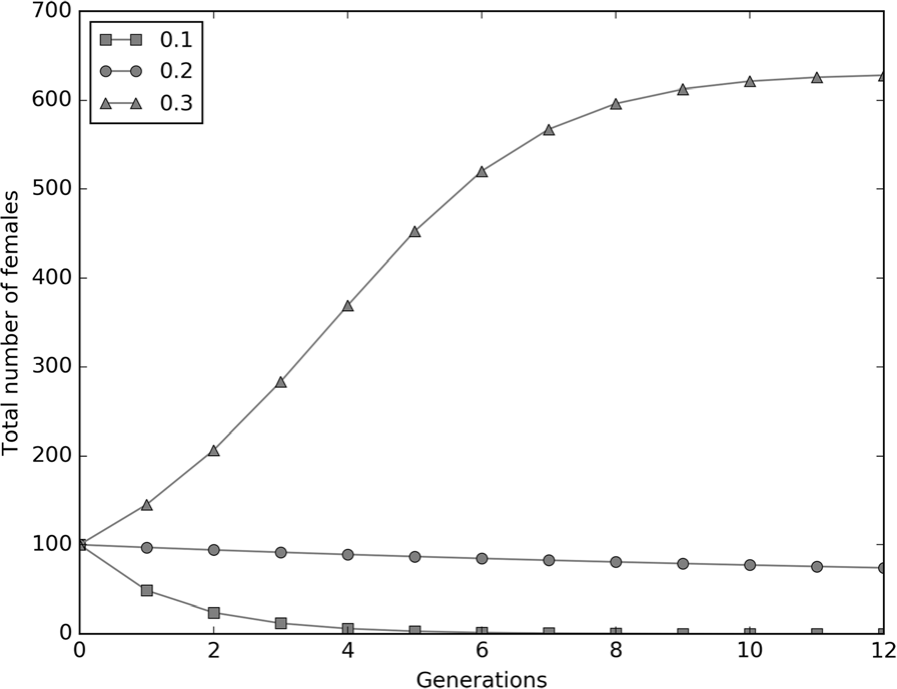
Simulation of the population dynamics on varying immature survival (S_0_) when incompatible infection types coexist at 1:1 ratio. The model simulation is relative to a mixed *Ae. albopictus* population constituted by two bidirectionally incompatible *Wolbachia* infection types at a ratio of 1:1. Mean immature survival for *Ae. albopictus* in nature is known to approach 20% [58]. Halving egg fertility by introducing bidirectional CI could make the population more susceptible to control measures targeting larval stage. Under the tested conditions, eradication became achievable by reducing immature survival to 10% and without requiring further incompatible male releases. S_0_ percent data are reported in legend as proportions

As shown in Fig. 5, immature survival may have a profound impact on the population dynamics. Reducing S_0_ to 10% was found to drive to eradication an *Ae. albopictus* population constituted by two *Wolbachia* infection types at a 50:50 ratio when they are bidirectionally incompatible. In fact, this CI pattern is already sufficient to halve egg hatch and, below a certain survival threshold, suppression would be self-sustaining and further incompatible male releases would not be required.

**Fig. 6:**
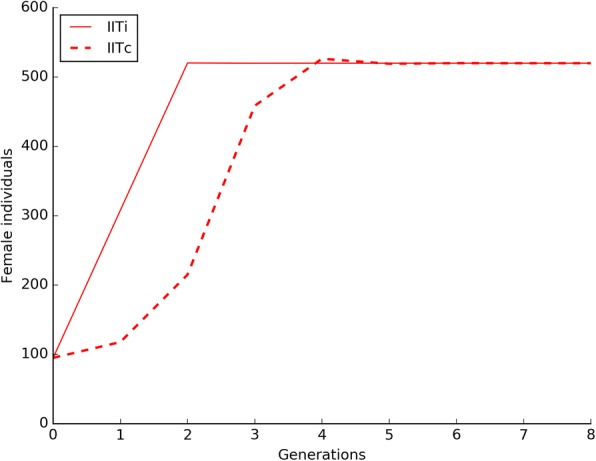
Simulation of the expected population dynamics when one or two *Wolbachia* infection types are present. The simulation shows the evolution of two systems subjected, respectively, to IIT_i_ and IIT_c_ after stopping incompatible male releases. As IIT_i_ releases are stopped, the natural population growth rate is suddenly restored. This outcome is common to all of the methods based on the release of sterile males. The IIT_c_ model simulation started with a mixed *Ae. albopictus* population constituted by two bidirectionally incompatible *Wolbachia* infection types at a ratio of 45:55. The control effects of the latter strategy last for several generations as incompatible males continue to be produced until the females of the infection type at lower frequency gradually extinguish

In the case of a 45:55 ratio, the infection type at higher frequency gradually caused the decline of the other one but the control effects of CI lasted for several generations because incompatible crosses continued to occur until the complete fixation of a single *Wolbachia* infection (Fig. 6). This phenomenon could occur when introducing an incompatible infection type by IIT_c_ and then stopping releases. In control simulation, possibly also representing the end of an IIT_i_ program, the natural population growth rate of the pure wild-type population is suddenly restored..

## Discussion

In this work, we adapted an available model describing the effect of *Wolbachia*-induced cytoplasmic incompatibility on host population size [29] to the specific case of an *Ae. albopictus* line with manipulated *Wolbachia* infection (AR*w*P) [44], aiming to test efficacy and safety of the Incompatible Insect Technique [18]. In fact, the females harboring the new *Wolbachia* infection, possibly released with the incompatible males, could mate and reproduce in the wild [42, 47] leading to consequences which need careful investigation [59]. Furthermore, in the case of establishment of the new infection type, the release of further incompatible males would lose any efficacy.

The risks associated with the release of mosquito vectors with manipulated *Wolbachia* infection have been widely analyzed and they are generally considered negligible when the infection determines a reduced vectorial competence [60–63]. However, each single case should be studied with specific attention to better understand the long-term evolution of the system [59].

In the case of unidirectional incompatibility patterns, theoretical models predict that repeated release of males and females harboring a suitable *Wolbachia* infection type might result in a progressive population replacement of the wild-types [35]. These expectations are being confirmed in the field with a *w*Mel *Wolbachia*-transinfected *Ae. aegypti* [28, 64, 65]. Also, models predict that the threshold over which replacement occurs is generally significantly higher when the conflict between infection types is regulated by a bidirectional incompatibility pattern. In fact, when bidirectionality is perfect and not significant fitness costs are associated to the *Wolbachia* infection, this threshold approximates a value of 50% of the frequencies [29]. This theoretical expectation was confirmed by our studies on AR*w*P *Ae. albopictus*. In fact, the presence of a few fertile females in a much larger population harboring the wild-type *Wolbachia* infection was proved not to lead to a population replacement. Instead, starting from a frequency of about 17%, the AR*w*P infection type extinguished in less than 4 generations.

However, while it is obvious that a single AR*w*P male release, even if contaminated by fertile AR*w*P females would not lead to a population replacement, releases carried out at short intervals and repeated for long periods may theoretically allow an AR*w*P population to locally increase in size before its natural decline. The IIT experiments demonstrated that an efficient suppression of a wild-type population of *Ae. albopictus* might be achieved by releasing AR*w*P males. These experiments were also useful to compare the results achievable when IIT was based on male-only or female-contaminated releases. In fact, the outcomes of these two strategies significantly diverged as the target wild-type population approached elimination. The results obtained in large cages could be generalized to an open field context taking into account that most of the assumptions employed in our model had to be discussed and adjusted to fit with a wider and more complex environment. Nevertheless, not all of our assumptions are expected to have a significant impact on the outcome of the simulation. For example, when releasing the incompatible males, the contaminant females would have a higher probability to mate with the co-released males harboring the same infection type due to their high density at the point of release. The fertility of these females could be partly diminished by further matings with the wild-type males [66] but it is certain that perfect random matings could not be assumed at releases. We assumed a 1:1 sex ratio and we kept it as a constant also in experiments but we know that sex ratio can be highly distorted in nature in dependence of the availability of food [55, 67] or as a consequence of a sex-specific differential susceptibility to insecticides [68]. The *Wolbachia* infection type is not known to affect sex ratio in AR*w*P *Ae. albopictus* [41], however it proved to induce significant effects on immature development as the time needed for AR*w*P pupation is significantly shorter compared to the S_ANG_ line, although the two lines share the same genetic background [46]. Even if this difference is only a few hours, we cannot exclude that it could furnish a further small advantage to AR*w*P over wild-type *Ae. albopictus* in the long term by contributing to a higher rate of population growth. However, results obtained under laboratory conditions could diverge significantly when testing the response of different *Wolbachia* infection types with a limited availability of resources for larval development, as common in open field [69]. These conditions certainly deserve further consideration as they could also highlight differences between *Wolbachia* infection types which could favor wild-type or AR*w*P individuals through delayed density-related effects (herein, *C*_*L*_ factor).

Models accounting for the overlapping of the generations [34] would be certainly more suitable for describing the effects of the CI on the population dynamics when the infection types determine differential effects on fitness or when CI is age-dependant. Nevertheless, validating these models experimentally would be far more complex and a series of assumptions would be required as well. Furthermore, apart from the reported small differences in immature development time, AR*w*P and S_ANG_ *Ae. albopictus* did not show significant difference with regard to fitness. Instead, we demonstrated that the low level of fertility shown by the wild-type males with respect to the AR*w*P females and the higher mating competitiveness index shown by AR*w*P males were already sufficient to reduce below 50% the frequency needed by AR*w*P to initiate a population replacement. This is why, in our experimental conditions, AR*w*P gains an advantage when the overall population approaches eradication or when the frequencies of the two populations are equal.

Thus, if it is certain that a local establishment of AR*w*P might occur in the case of a nearly complete eradication of *Ae. albopictus* from an area, further considerations are needed to estimate the possibility of spatial spread of the AR*w*P infection type when moving from a confined environment to an experimental system open to immigration and emigration of individuals [70]. According to mathematical models, migration tends to decrease the *Wolbachia* invasion rate even in the case of unidirectional incompatibility patterns [34]. This expectation has been confirmed with *Ae. aegypti* and other mosquito species [69, 71], also due to possible fitness advantages characterizing the wild-type strains [72]. This limit to the spatial spread would be clearly stronger with bidirectional incompatibility patterns. It is possible that, once locally established, a small AR*w*P *Ae. albopictus* population could survive for a while, due to the nearly island-like distribution of *Ae. albopictus* [73]. However, breeding sites are not supposed to be stable in the long term and the associated metapopulations are known to be interconnected by migratory fluxes and passive transport [74, 75]. Therefore, being outnumbered by the wild-type *Ae. albopictus*, the AR*w*P population would be expected to gradually extinguish because no stable equilibrium exists in panmictic populations in which there are two incompatible crossing types [76]. Our model simulation clearly highlighted this outcome by testing the effect of migration at low population turnover rates.

If we hypothesize area-wide IIT programs or programs aiming at *Ae. albopictus* eradication in isolated regions such as islands or newly colonized localities, it is clear that the risk of population replacement has to be taken into account seriously. In the latter case, using SIT or strategies combining IIT and irradiation [16, 47, 77] could be an option to avoid any risk of replacement and loss of efficacy of the control strategy. The same approaches would be strictly required when releasing *Wolbachia* transinfected lines characterized by unidirectional CI patterns and showing equal or increased vector competence for severe diseases endemic in the target area.

Besides the perceivable risks highlighted above, our studies put in evidence advantages specific to the IIT strategy based on bidirectional CI which could be exploited in certain circumstances, in particular when operational programs may exploit mosquito strains rigorously proven to be attenuated vectors. Other authors have already pointed out that a single massive release of females harboring a *Wolbachia* infection type and showing bidirectional incompatibility with the wild-types would result in multiple generations of suppression of the target population because released females would continue to produce incompatible males until the elimination of one the two infection types [29]. This expectation was also confirmed by our model when comparing the outcome of IIT_i_ and IIT_c_ approaches in the case of interruption of the releases. However, such strategy is not likely to be approved due to the level of nuisance and risk of disease transmission it would cause.

As an alternative, represented by the IIT_c_ approach, small percentages of contaminant females could be released together with the incompatible males aiming at obtaining a strong suppression effect due to the released males in the short-term and a gradually increasing long-term suppression effect due to the increase of the frequency of the females of the new line. Importantly, this gradual increase in the frequencies of the new infection type would not be self-sustaining as it will be strictly dependent on the continuous release of new females until the achievement of the required threshold to obtain a replacement by the released infection type. Thus, replacement risks could be managed. As such, introducing bidirectional CI as a self-sustaining factor of sterility into the wild-type mosquitoes is expected to reduce the population growth rate and make the target vectors more vulnerable to other components of an Integrated Vector Management approach [78] also including larval control, as evidenced by Fig. 5.

In the case of need, managing CI [29] would require a periodical survey of the infection frequencies. which could be performed by diagnostic PCR, by checking the mean egg fertility (which is expected to be halved at nearly equal frequencies between the two infection types) or combining these two approaches aiming at saving costs. Thus, a rigorous cost/benefit analysis should be performed to compare this approach with the available alternatives in terms of efficacy and sustainability in the long term.

Further research will be needed to test the discussed results regarding AR*w*P *Ae. albopictus* by pilot trials in semi-field and field conditions. Particular attention will be paid to study density dependent demographic traits, operating during larval stage, which could be key factors in determining the outcome of area-wide programs [72]. These studies will also be addressed to the determination of the *C*_*L*_ factor, as defined in the Methods section, to refine the model that we are using to support our experiments on AR*w*P. An additional *Wolbachia*-transinfected line of *Ae. albopictus* which shows strongly reduced vector competence for chikungunya, dengue and Zika viruses has been recently established at ENEA and it will be tested as a possible alternative to AR*w*P [79].

## Conclusions

In this work, the Incompatible Insect Technique based on bidirectional incompatibility was tested as a tool for controlling *Ae. albopictus* by coupling laboratory trials with a modelistic approach. Our study confirmed the potential effectiveness of this control method and investigated key safety and sustainability issues. In particular, IIT_c_ could head towards population suppression or population replacement strategies depending on the context and on the availability of vector strains with suitable *Wolbachia* infection types. This groundbreaking view might allow us to make the most of *Wolbachia* properties for maintaining a reduced level of egg fertility on a longer term, also due to the coexistence of two incompatible populations. The costs for sterile males production could be also more sustainable with respect to other approaches as sexing protocols could be less severe and males to be released could be reduced. Population replacement purposes could be pursued locally in the case of availability of *Wolbachia* infection types capable of significantly attenuating the vector competence of the target species.

## Additional files

Additional file 1: **Table S1.** Female fecundity and immature survival in AR*w*P and S_ANG_ *Ae. albopictus* (*m* and *S*_*0*_ values). (DOC 31 kb)

Additional file 2: **Table S2.** Calculation of the mean level of egg fertility expected in CI crosses between AR*w*P females and S_ANG_ males (= *H*_*X*_ value). (DOC 34 kb)

Additional file 3: **Table S3.** Calculation of the mean level of male mating competitiveness of AR*w*P compared to S_ANG_ *Ae. albopictus* males. (DOC 33 kb)

Additional file 4: **Figure S1.** Model simulation of the IITi experiment showing how the outcome of the population dynamics is influenced by the variation of *γ* and *α* parameters. (PDF 200 kb)

## Abbreviations

AR*w*P: *w*Pip *Wolbachia* infected *Ae. albopictus* sharing the same genetic background with S_ANG_
CHIKV: Chikungunya virus
CI: Cytoplasmic incompatibility
DENV: Dengue virus
ENEA: Italian National Agency for New Technologies, Energy and Sustainable Economic Development
IIT: Incompatibe insect technique
IIT_c_: IIT with contaminant females among released males
IIT_i_: IIT without contaminant females among released males
S_ANG_: Wild-type *Aedes albopictus* from Anguillara Sabazia (Rome province, Italy) with natural *Wolbachia* superinfection
SIT: Sterile insect technique
ZIKV: Zika virus
