# Supplementary material for: Cytoplasmic incompatibility management to support Incompatible Insect Technique against Aedes albopictus
Source: Parasit Vectors. 2018 Dec 24;11(Suppl 2):649. doi: 10.1186/s13071-018-3208-7 (PMC6304776; doi:10.1186/s13071-018-3208-7)
Supplement: Supplementary file 1 — Table S1. Female fecundity and immature survival in ARwP and SANG Ae. albopictus (m and S0 values). (DOC 31 kb) [file 13071_2018_3208_MOESM1_ESM.doc]

**Additional file 1: Table S1**

| **Table S1.** Female fecundity and immature survival in AR*w*P and S_ANG_ *Ae. albopictus* | | |
| --- | --- | --- |
|  | *m* | *S_0_* |
| S_ANG_ | 19.87 ± 3.56 | 85.01± 3.96 |
| AR*w*P | 19.89 ± 3.96 | 84.81 ± 3.52 |
| S_ANG_ = wild-type *Ae. albopictus* with natural *Wolbachia* infection; AR*w*P = *w*Pip *Wolbachia* infected *Ae. albopictus*; *m* = mean female L_1_ per female; *S_0_* = percentage of first instar larvae reaching adult stage;  Within columns, results were not significantly different by ANOVA (*P* > 0.05); *m: F* = 0.03; *df* = 8; *P* = 0.86; *S_0_: F* = 0.01; *df* = 8; *P* = 0.92 | | |

**Methods**: S_ANG_ and AR*w*P populations consisting of 3-7 days old 50:50 females:males were allowed to mate in 100 ×100×100 cm cages for 24 hours and then provided with a blood meal. Produced eggs were collected on wet germination paper until 7^th^ day after feeding, counted and then hatched to measure female fecundity and mean egg hatch rate. The obtained first instar larvae were used to prepare rearing trays each containing 200 larvae in 200 ml of water and fed as previously described until pupation. Adults were then counted to measure the immature survival to be compared between lines. Each treatment was replicated five times.
