# Supplementary material for: Cytoplasmic incompatibility management to support Incompatible Insect Technique against Aedes albopictus
Source: Parasit Vectors. 2018 Dec 24;11(Suppl 2):649. doi: 10.1186/s13071-018-3208-7 (PMC6304776; doi:10.1186/s13071-018-3208-7)
Supplement: Supplementary file 2 — Table S2. Calculation of the mean level of egg fertility expected in CI crosses between ARwP females and SANG males (= HX value). (DOC 34 kb) [file 13071_2018_3208_MOESM2_ESM.doc]

| **Table S2**. **Calculation of the mean level of egg fertility expected in CI crosses between AR*w*P females and S_ANG_ males (= *H_X_*value).** The mean egg fertility when S_ANG_ males are not infected by *w*AlbA *Wolbachia* was measured previously^a^. The mean percentage of S_ANG_ males which were not *w*AlbA infected was measured by sampling 10 males, obtained by following the rearing conditions applied in the experiments reported herein and aged 4 ± 1 days, per each one of five experimental cages. | | |
| --- | --- | --- |
| Mean egg fertility when S_ANG_ males are not infected by *w*AlbA *Wolbachia^a^* | Mean percentage of S_ANG_ males not *w*AlbA infected | mean *H_X_* value expected in our experiments |
| 31.94 ± 6.20 | 8.00 ± 2.58 | 2.56 ± 0.02 |
| ^a^S_ANG_ males were considered not infected by *w*AlbA *Wolbachia* when the *w*AlbA titer is <0.001 *w*AlbA/actin copy numbers by qPCR (Calvitti et al., 2015). | | |
