# Supplementary material for: Cytoplasmic incompatibility management to support Incompatible Insect Technique against Aedes albopictus
Source: Parasit Vectors. 2018 Dec 24;11(Suppl 2):649. doi: 10.1186/s13071-018-3208-7 (PMC6304776; doi:10.1186/s13071-018-3208-7)
Supplement: Supplementary file 3 — Table S3. Calculation of the mean level of male mating competitiveness of ARwP compared to SANG Ae. albopictus males. (DOC 33 kb) [file 13071_2018_3208_MOESM3_ESM.doc]

| **Table S3**. **Calculation of the mean level of male mating competitiveness of AR*w*P compared to S_ANG_ *Ae. albopictus* males.** Experimental cages were prepared according to the same experimental conditions described in the manuscript. S_ANG_ wild-types and AR*w*P males were released in the cages at a ratio of 50:50. Then 50 S_ANG_ females were added. A blood meal was provided and produced eggs collected to measure egg fertility. A male competitiveness index (Fried index) was measured according to previous methods by comparing the obtained results with those expected in CI crosses and S_ANG_ fertile crosses (Moretti and Calvitti, 2013). The experiment was repeated three times. | | | | | |
| --- | --- | --- | --- | --- | --- |
| Mean (±SE) egg fertility in S_ANG_ fertile crosses (S_ANG_ females × S_ANG_ males) | Mean (±SE) egg fertility in CI crosses (S_ANG_ females × AR*w*P males) | Mean (±SE) observed egg fertility in competition trials | Fried index of male mating competitiveness for AR*w*P males^a^ | Mating competitiveness of AR*w*P males relative to the S_ANG_ males^b^ | Mating competitiveness of S_ANG_ males relative to the AR*w*P males^b^ |
| 75.62 ± 6.95 | 0.00 ± 0.00 | 28.76 ± 2.25 | 1.63 | 1.24 | 0.76 |
| ^a^measured by considering S_ANG_ male mating competitiveness as = 1;  ^b^calculated by considering S_ANG_ + AR*w*P male mating competitiveness = 2. | | | | | |
