# Supplementary material for: Cytoplasmic incompatibility management to support Incompatible Insect Technique against Aedes albopictus
Source: Parasit Vectors. 2018 Dec 24;11(Suppl 2):649. doi: 10.1186/s13071-018-3208-7 (PMC6304776; doi:10.1186/s13071-018-3208-7)
Supplement: Supplementary file 4 — Figure S1. Model simulation of the IITi experiment showing how the outcome of the population dynamics is influenced by the variation of γ and α parameters. (PDF 200 kb) [file 13071_2018_3208_MOESM4_ESM.pdf]

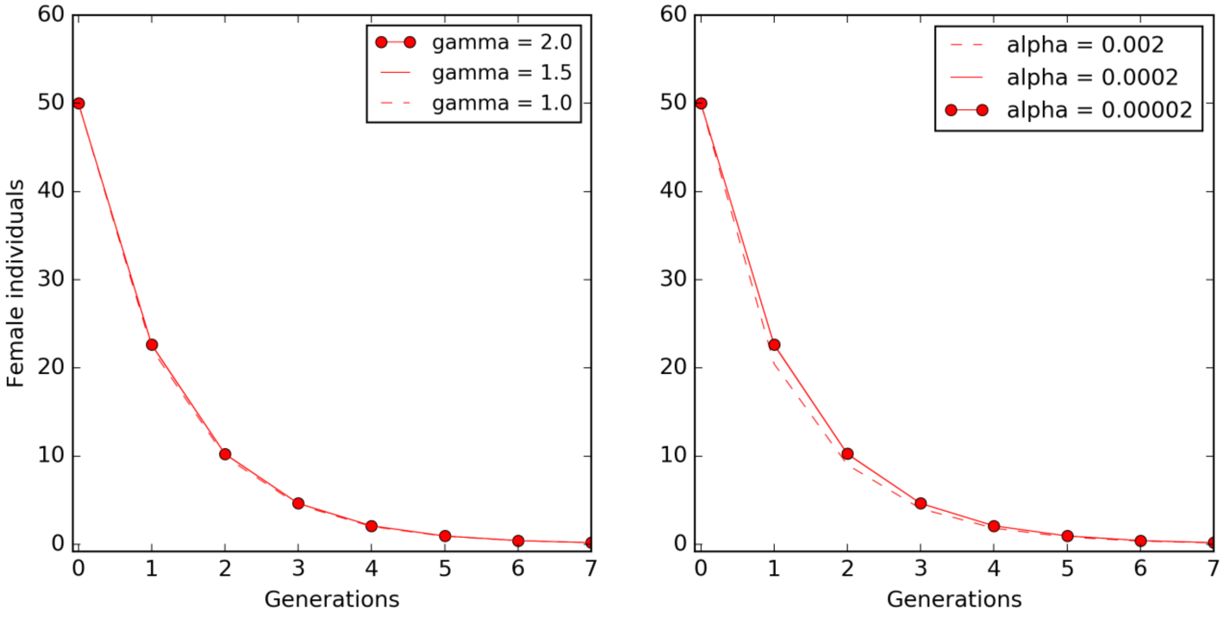

Figure S1. Model simulation of the IIT$_i$ experiment showing how the outcome of the population dynamics is influenced by the variation of γ and α parameters.
